# Combining Biochemical and Imaging Markers to Improve Diagnosis and Characterization of Mild Traumatic Brain Injury in the Acute Setting: Results from a Pilot Study

**DOI:** 10.1371/journal.pone.0080296

**Published:** 2013-11-19

**Authors:** Zhifeng Kou, Ramtilak Gattu, Firas Kobeissy, Robert D. Welch, Brian J. O’Neil, John L. Woodard, Syed Imran Ayaz, Andrew Kulek, Robert Kas-Shamoun, Valerie Mika, Conor Zuk, Francesco Tomasello, Stefania Mondello

**Affiliations:** 1 Department of Biomedical Engineering, Wayne State University, Detroit, Michigan, United States of America; 2 Department of Radiology, School of Medicine, Wayne State University, Detroit, Michigan, United States of America; 3 Center for Neuroproteomics and Biomarkers Research, Department of Psychiatry, McKnight Brain Institute, University of Florida, Gainesville, Florida, United States of America; 4 Department of Emergency Medicine, School of Medicine, Wayne State University, Detroit, Michigan, United States of America; 5 The Cardiovascular Research Institute, School of Medicine, Wayne State University, Detroit, Michigan, United States of America; 6 Department of Psychology, Wayne State University, Detroit, Michigan, United States of America; 7 Department of Neurosciences, University of Messina, Messina, Italy; University of Illinois at Chicago, United States of America

## Abstract

**Background:**

Mild traumatic brain injury (mTBI) is a significant healthcare burden and its diagnosis remains a challenge in the emergency department. Serum biomarkers and advanced magnetic resonance imaging (MRI) techniques have already demonstrated their potential to improve the detection of brain injury even in patients with negative computed tomography (CT) findings. The objective of this study was to determine the clinical value of a combinational use of both blood biomarkers and MRI in mTBI detection and their characterization in the acute setting (within 24 hours after injury).

**Methods:**

Nine patients with mTBI were prospectively recruited from the emergency department. Serum samples were collected at the time of hospital admission and every 6 hours up to 24 hours post injury. Neuronal (Ubiquitin C-terminal Hydrolase-L1 [UCH-L1]) and glial (glial fibrillary acidic protein [GFAP]) biomarker levels were analyzed. Advanced MRI data were acquired at 9±6.91 hours after injury. Patients’ neurocognitive status was assessed by using the Standard Assessment of Concussion (SAC) instrument.

**Results:**

The median serum levels of UCH-L1 and GFAP on admission were increased 4.9 folds and 10.6 folds, respectively, compared to reference values. Three patients were found to have intracranial hemorrhages on SWI, all of whom had very high GFAP levels. Total volume of brain white matter (WM) with abnormal fractional anisotropy (FA) measures of diffusion tensor imaging (DTI) were negatively correlated with patients’ SAC scores, including delayed recall. Both increased and decreased DTI-FA values were observed in the same subjects. Serum biomarker level was not correlated with patients’ DTI data nor SAC score.

**Conclusions:**

Blood biomarkers and advanced MRI may correlate or complement each other in different aspects of mTBI detection and characterization. GFAP might have potential to serve as a clinical screening tool for intracranial bleeding. UCH-L1 complements MRI in injury detection. Impairment at WM tracts may account for the patients’ neurocognitive symptoms.

## Introduction

Traumatic brain injury (TBI) is a significant public healthcare burden in the United States, accounting for 1.7 million incidents in the United States each year [1,2]. The majority of TBI patients belong to the mild TBI (mTBI) severity group due to the improvement of motor vehicle safety design in recent years [3]. mTBI leads to over 1 million emergency visits in the United States each year [4]. It causes a constellation of physical, cognitive, and emotional symptoms that significantly impact the patients’ quality of life, and costs the nation $16.7 billion each year [3,5,6]. Currently, most mTBI patients stay in the emergency department (ED) for only a few hours and then are discharged home without a concrete follow-up plan or an understanding of potential symptoms. Up to 50% of mTBI patients develop neurocognitive problems within the first month [7,8], and 5-15% of them continue to manifest neurocognitive sequelae at one year [7,9]. Often, their neurocognitive outcomes inconsistently correlate with clinical measures such as the Glasgow Coma Scale (GCS) score and post-traumatic amnesia. Most mTBI patients do not have abnormalities on computed tomography (CT) and conventional magnetic resonance imaging (MRI) [10,11] in the emergent setting. The immediate challenge for emergency physicians is to identify intracranial abnormalities in those CT-negative patients, who may have long-term neurocognitive symptoms [12]. 

Advanced MRI has demonstrated improved sensitivity in detecting TBI pathologies and functional impairments that underlie patients’ cognitive symptoms [13,14]. Examples include diffusion tensor imaging (DTI) of axonal injury [15-24], susceptibility weighted imaging (SWI) of hemorrhagic lesions [25-27], and others. Advanced MRI offers anatomical and pathological information, reflecting brain damage with high spatial resolution [13,14]. The American College of Emergency Physicians and Center for Disease Control Joint Study Panel highly recommended examining the role of advanced MRI in the acute setting (within 24 hours after injury) [12]. However, to date, very few studies have scanned mTBI patients within 24 hours after injury when the patients are still in the ED. This is mainly due to, first the high cost of MRI making it prohibitive to scan all mTBI patients, and second the inability to access the MRI acutely 24/7. Consequently, in the majority of medical centers in North America, MRI is not a standard of care of mTBI patients in the acute setting. 

In addition to patient’s clinical characteristics and advanced neuroimaging studies, brain–specific proteins released into the bloodstream after brain injury, as a result of cellular damage and activation, have demonstrated the potential to serve as diagnostic and prognostic markers in mTBI [28,29]. Along with providing improved diagnostic capability and molecular characterization of subjects who sustained mTBI, appropriate biomarker screening may lead to a more selective strategy for neuroimaging, reducing the need for a substantial number of unnecessary imaging exams. With these aims in mind, several groups recently reported the application of neuroproteomics to identify and characterize biochemical markers of TBI [30]. Among the variety of biomarkers that have been used to investigate the neuronal marker, Ubiquitin C-terminal Hydrolase-L1 (UCH-L1) [31-33] and the astrocyte-specific protein Glial Fibrillary Acidic Protein (GFAP) [34], and αII-Spectrin Breakdown Products (SBDPs) for axonal injury [35,36] seem particularly promising. 

UCH-L1 is a small (25 kDa), neuronal protease involved in either the addition or removal of ubiquitin from proteins that are destined for metabolism via the ATP-dependent proteasome pathway; it is highly enriched in the brain (1 - 5% of total soluble brain protein)[37,38]. Mutations and polymorphisms of UCH-L1 have been associated with familial Parkinson’s Disease [39]. UCH-L1 is released into the extracellular space as a consequence of cell destruction under diverse pathological conditions affecting the brain. Previous clinical studies have demonstrated increased UCH-L1 levels in cerebral spinal fluid (CSF) and in serum in severe TBI patients and that the magnitude of this increase correlated with injury severity, CT finding and patient outcome [31,33]. Recently, a study was completed investigating UCH-L1 in adults with mild and moderate TBI showing increased UCH-L1 levels in mTBI patients compared to uninjured controls and that UCH-L1 was able to detect intracranial lesions on CT with an area under the curve (AUC) of 0.73 [40]. Based on these encouraging results and the fact that UCH-L1 is specific to neurons and its high specificity and abundance in the CNS, it appears to be an excellent candidate biomarker for the brain injury clinical studies. 

Compared with UCH-L1, GFAP is a monomeric intermediate filament protein, a major constituent of the astroglial cytoskeleton, and highly brain-specific [41,42]. This glial protein represents an ideal complement of the neuronal UCH-L1, as demonstrated by a recent study showing that the correlations between these 2 markers reflect structural changes detected by neuroimaging and may be used as an indicator for differing intracranial pathologies after brain trauma [43]. Additionally, previous studies evaluating severe TBI patients demonstrated that GFAP concentrations were associated with injury severity and outcome [44,45]. Recently, in a prospective cohort study of 108 patients with mild or moderate TBI, GFAP was found to be elevated in the serum within 1 h after injury, discerning TBI patients from uninjured controls with an area under the curve (AUC) of 0.90 and discriminating patients with and without intracranial lesion as assessed by CT with an AUC of 0.79 [46]. The same study also reported a significant difference in GFAP levels between mild TBI (GCS 15) and general trauma controls [47], in suggestion of the CNS-specific nature of GFAP. 

As a simple biofluid-based rapid diagnostic tool, serum biochemical markers offer great potential for rapid, accurate, and cost-effective diagnosis of brain injury, and a temporal profile of blood protein levels might be indicative of disease progression or resolution. Particularly, several biochemical markers, including UHC-L1 and GFAP, among others, are reported being CNS-specific, which makes them ideal for brain injury detection. On the other hand, emerging data suggest that MRI and especially advanced MRI techniques are very sensitive in detecting brain injury that are occult in clinical imaging by providing spatial and pathophysiological information. Although the combinational and complementary use of these tools is promising and might have important implications for improving injury detection and outcome prediction, the correlation among injury pathologies at tissue level assessed by neuroimaging and at the protein level as assessed by blood biomarker profiles has not yet been elucidated. 

We hypothesized that, in mTBI patients, elevated GFAP levels will be associated with intracranial abnormalities detected by baseline MRI and SWI, and elevated UCH-L1 will be associated with white matter damage indicated by DTI. Our objective in this study was to evaluate serum GFAP and UCH-L1 levels after mTBI and their correlation to the advanced MRI findings, including SWI and DTI, in a pilot cohort in an acute setting. In particular, we were interested in establishing a serum profile of these biomarkers that might serve as signatures for the presence of brain pathology as assessed by advanced MRI methods, and thus aid in the identification of patients who need an MRI scan in the acute setting.

## Materials and Methods

### Patient Recruitment

This study was approved by both the Human Investigation Committee of Wayne State University and the Institutional Review Board of Detroit Medical Center. Written informed consent was obtained from each subject before enrollment.

A total of 9 patients who sustained mTBI were prospectively recruited from the ED of Detroit Receiving Hospital (DRH), a Level-1 trauma center, which is an affiliated hospital of Detroit Medical Center (DMC). Patient eligibility was based on the mTBI definition by the American Congress of Rehabilitation Medicine [48] with the following inclusion criteria: Patients aged 18 or older with an initial Glasgow Coma Scale (GCS) score of 13-15 in ED with any period of loss of consciousness less than 30 minutes or any post traumatic amnesia less than 24 hours, or recorded change of mental status (confused, disoriented or dazed). Patients with a GCS of <15 were screened with the Conley test for the ability to consent. All patients required a CT scan as part of their clinical evaluation. All of them were be able to speak English. The exclusion criteria included patients under the age of 18 years, pregnant woman, medically documented history of brain injury, neurological disorders or psychoactive medications, history of substance abuse, CT indication of any metal in the brain and body, known contraindication to MRI such as a pacemaker or other non-MR compatible implanted device as defined by metal screening procedure, or patients without a clear history of trauma as their primary event (e.g., seizure, epilepsy, etc). In the acute stage, a patient might have mental status change or amnesia in which medical history may not be properly obtained, thus the patient's record was retrospectively screened as well to exclude any patient who does not fit our inclusion criteria. Additionally, we performed an imaging study of 18 healthy controls without history of head injury or antecedents of central nervous system disease. 

### Neurocognitive Assessments

At the acute setting, once a patient was conscious and stable, they were administered neurocognitive tests and surveyed about their post-concussion symptoms (PCS). Given the situation of emergency care, it is not feasible to perform a full battery of neuropsychological assessment. Instead, a short instrument called Standardized Assessment of Concussion (SAC) [49] was used to assess the patients’ neurocognitive status. The SAC instrument was originally developed for onsite testing of subject’s neurocognitive performance after sports concussion [50]. It has been reported that SAC is sensitive to the acute changes following concussion and it only requires limited training of an administrator [51]. The SAC assesses 4 cognitive domains including orientation, attention, immediate memory and delayed recall, and the resulting points give a patient score between 0 (indicating greater cognitive deﬁcit) and 30. Previous results report its sensitivity to brain injury in the emergency setting, particularly in that delayed recall is more sensitive to brain injury [51]. The Emergency Room Edition of the SAC instrument also has a graded symptom checklist with all PCS symptoms listed. The patients were asked to grade each symptom from none, mild, moderate, to severe, (graded from a 0 to 3 respectively). The total points were the overall PCS score.

### Neuroimaging Protocol

In the ED, once a patient was cleared of any immediate life-threatening risk following a CT scan and was stable enough for an MRI, the patient was transported to the MRI center for imaging scan. All MRI data were collected on a 3-Tesla Siemens Verio scanner with a 32-channel radio frequency head coil. The subject’s head was fixed by a foam pad to restrict motion. Imaging protocol included SWI, DTI, and resting state functional MRI, in addition to the baseline structural imaging (T1, T2 gradient recalled echo [GRE] and T2 fluid-attenuated inversion recovery [FLAIR]) sequences, with total data acquisition time of 39 min. Of the 9 patients, resting state fMRI data was collected in decent quality for only 4 of them. The rest of the patients either could not stay in the magnet any longer after baseline MRI and SWI and DTI sequences or did not cooperate for resting state data collection. Therefore, this paper focuses on the relationship between blood biomarkers and SWI and DTI data.

SWI is a 3-dimentional, T2* based GRE sequence with long TE and 3-D flow compensation. The phase images were high-pass filtered (96x96 filter size) by using an in-line manufacture-applied filter and then integrated with magnitude images to generate the processed SWI image to better delineate the spatial relation between microhemorrhages and veins [13,52]. SWI parameters include TR/TE of 30/20ms, flip angle of 15 degree, bandwidth of 100 Hz/Px, field of view (FOV) of 256x256 mm^2^, imaging matrix of 512x256, 25% oversampling, slice thickness of 2 mm, total 64 slices, 20% distance factor, GRAPPA iPat factor of 2, with resultant voxel size of 0.5x1x2 mm^3^ and imaging acquisition time 4m and 18s. 

DTI sequence is a standard echo planar imaging (EPI) 2D sequence provided as part of the Siemens package. The parameters include TR/TE of 13300/124 ms, EPI factor of 192, bandwidth of 1240 Hz/Px, FOV of 256x256 mm^2^, imaging matrix of 192x192, slice thickness of 2 mm, total 60 slices, no gap between slices, 30 gradient directions, 2 averages, B values of 0/1000 s/mm^2^, anterior-posterior phase encoding, GRAPPA acceleration factor of 2, with resultant voxel size of 1.3x1.3x2 mm^3^ and data acquisition time of 14m 26s. 

### Image Processing and Interpretation

All SWI and DTI images were processed by a co-author who was blinded to the subjects’ clinical conditions to avoid any bias. All SWI images were further processed by using our in-house software SPIN (signal processing for NMRI) (MRI Research Institute, Detroit, Michigan). All structural MRI images, including SWI images, were read by two board certified neuroradiologists to identify other conditions that may confound the findings. The neuroradiogists were blinded to the medical history and conditions of subjects to avoid any bias as well. We also graded the structural imaging findings based on their radiologic report for statistical analysis: 0 for negative finding; 1 for non-specific finding, including non-specific WM hyperintensities; and 2 for traumatic hemorrhage. 

#### DTI image processing

Given the relatively long sequence in our DTI data collection (2 averages, total 14m 26s), several steps were taken to overcome the potential motion artifacts: 1) During every scan, each subject’s head was tightly enclosed within the head coil with soft padding in a comfortable position; 2) Instead of inline averaging of two volumes of data, each volume was saved individually for later motion correction; 3) A motion correction and eddy current correction algorithm, automatic image registration (AIR) [53], was applied to correct all the diffusion-weighted images for each volume of data by using B0 image as the reference image; and, finally, 4) two volumes of motion-corrected images were averaged for further preprocessing.

Preprocessing of DTI images was carried out by using DTI Studio (https://www.dtistudio.org). Fractional Anisotropy (FA) maps were created from the tensor calculations by suppressing the background noise on B0 image in DTI Studio. Skull stripping of the B0 image was done by using the BET routine package in Mricro with a fractional intensity of 0.1. A binary mask of the skull-stripped B0 image was used on the FA map to preserve the pure brain parenchyma. 

#### Voxel based analysis (VBA)

VBA was performed after the skull stripped FA image was spatially normalized, by using a non-linear algorithm, to the standard FMRIB FA template for all the 27 subjects (9 mTBI patients and 18 healthy controls) in SPM8 software 

(http://www.fil.ion.ucl.ac.uk/spm/software/spm8/). A mean FA map and a standard deviation of FA map were created for the controls. A Z-score map was created for each individual patient in a voxel-based approach: For each voxel, the FA difference between each individual patient and the mean of controls was divided by the standard deviation of the controls at the same voxel. Voxels with Z-score > 2 for increased FA and Z-score < -2 for decreased FA were selected for further consideration. 

#### Tract based spatial statistics (TBSS)

A similar approach was also used to evaluate the lesion load by using the TBSS method after registering each subject nonlinearly to the standard FMRIB FA template. The TBSS analytical approach was used to compare patient and control groups to evaluate the WM changes after TBI. All the processing steps were performed according to the TBSS manual (http://fsl.fmrib.ox.ac.uk/fsl/fslwiki/TBSS/UserGuide). Briefly, every subject’s FA image was spatially and non-linearly normalized to a standard FA FMRIB template and transformed into a standard space using FNIRT algorithm from FSL software package. Subsequently, a mean FA image was created from this set of non-linearly transformed images. A search algorithm then created a mean skeleton, looking for the local maxima perpendicular to the WM tract across the whole brain volume in all the transformed images, and then projected this skeleton across all the subjects in the group to extract the skeleton of individual subject. Next, a voxel-wise permutation-inference analysis was carried out between the skeletons of two groups, and a two tailed t-statistics was performed to extract the voxels that fall below or above a certain threshold. These voxels were converted to a p value based on the threshold set by the t-stat value and the cluster size. In TBSS analysis, a skeleton threshold of 0.3 was used to eliminate grey matter (GM) voxels or partial volume effects, and cluster forming threshold t of 4 was used.

#### Masking out non-white matter voxels

The selected abnormal voxels were further filtered to eliminate the GM and the non-WM voxels accounting for the partial volume effects arising from CSF and GM. This filtering was done by segmenting each non-linearly normalized FA map and the mean FA map from the controls into WM, GM and CSF in SPM8 and consequently creating a compound mask by applying a threshold (p>0.78) on these two segmented WM FA images. In this way, we were able to get rid of the false positives arising on the edges of WM because of the mis-registration, and voxels having FA < 0.3 were considered to be non WM voxels and discarded by using a mask. Spurious voxels that doesn’t form a cluster size of at least 10 voxels were discarded as random noise in VBA and cluster size of less than 5 were discarded as random noise in TBSS. Clusters were extracted using the cluster tool in FSL. As a result, the total number of selected voxels was defined as the lesion load for each subject in both VBA and TBSS analyses.

### Blood Collection and Biomarker Analysis

All blood samples were collected within 6 hours after injury, beginning upon subject’s arrival to ED and then every 6 hours thereafter, until discharge or up to 24 hours. Samples were immediately centrifuged at 4000 rpm for 10 min and frozen and stored at -80°C until the time of analysis. Blinded sample analysis was conducted in a central laboratory (Banyan Biomarkers, Alachua, FL) employing electro-chemiluminescent immunoassay method (ECL-IA) for quantitative analysis of UCH-L1 and GFAP in human serum samples using the MSD platform (MesoScale Discovery, Gaithersburg, MD). The UCH-L1 assay system utilizes a mouse monoclonal IgM anti-human UCH-L1 antibody for solid phase immobilization to capture UCH-L1 from samples. The UCH-L1 antigen in turn binds to a sulfo-tag labeled anti-mouse antibody. The GFAP ECL-IA utilizes a proprietary mouse monoclonal IgG anti-human GFAP antibody for solid phase immobilization and a proprietary polyclonal rabbit antibody for detection. The rabbit IgG polyconal detection antibody in turn binds to a sulfo-tag labeled anti-rabbit antibody. Detection signal can be measured when an electrical current is applied to the electrodes at the bottom of each well of the plate. The signal is measured at 620 nm. Quantitative determination of the biomarker concentration is achieved by comparing the unknown sample signal intensities to a standard curve, obtained from the calibrators run in the same assay. Target concentrations are reported in ng/ml. Each assay plate included 3 QC controls at high, medium and low concentrations of the assay range, each plated in duplicate. Calibrators were prepared in Pooled Human Serum (PHS) matrix. Specifically, a serial dilution of the calibrator protein is prepared and aliquots of that calibrator solution are assayed in the same assay volume and under the same conditions as the samples. The calibrator signal intensities were used to generate a dose-response curve and to calculate the sample concentrations using a weighted four-parameter logistic function (MSD software and MSD reader). The lower limit of detection of the UCH-L1 and GFAP assays was determined to be 0.10 and 0.008 ng/mL, respectively. Samples with undetectable levels of UCH-L1 or GFAP were assigned a value of 50% of the lower limit of detection (ie, 0.05 and 0.004 ng/mL, respectively). The median (IQR) serum UCH-L1 and GFAP concentrations determined in blood samples from 29 healthy volunteers recruited as part of an ongoing biomarker study were used as normal reference values. Healthy subjects were age and gender matched with TBI patients.

### Statistical analysis

Statistical analyses were performed by using SAS version 9.2 (Cary, NC, USA) and R software (http://www.r-project.org). Data normality was assessed by using the Kolmogorov–Smirnov test. Results for continuous variables are presented as mean (SD) or median (interquartile range) as appropriate. Frequencies and percentages are presented for categorical variables. Between-group differences were assessed by the Student’s t-test (for normally distributed continuous variables) and the Mann–Whitney U test (non-normal continuous variables). Pearson's chi-squared test was used to explore the relationships between categorical variables. Pearson correlations were performed to determine the relationships among different parameters, including imaging, biomarkers and patients neurocognitive measurements. The relationship between biomarker concentration and parameters for TBI severity, neuroimaging and neurocognitive scores was assessed by bivariate correlations (Spearman’s). Two sided tests were used and p<0.05 was considered significant.

## Results

### Characteristics of the Subjects

Individual patient demographic and clinical characteristics are presented in Table 1. In our mTBI cohort (n = 9), with 8 (89%) male subjects and 1 (11%) female subject, the average patient age was 41.22±14.37 (mean ± standard deviation) years, and all of their GCS scores were 15 upon ER admission. All patients had various length of loss of consciousness (LOC). Five (55%) patients were injured in assault and 4 (45%) were victims of motor vehicle accidents. The median SAC and PCS scores were 23.5 and 15.5, respectively. Two patients presented positive findings in acute CT scan: one with epidural hematoma and cortical contusion in the parieto-temporal region and the other with a small subarachnoid hemorrhage (SAH). MRI scans were performed at 9 ±6.91 hours after injury. Three patients, including those two CT positive patients, presented hemorrhagic findings on structural MRI. One of these patients (Case 1) presented small hemorrhages that were completely missed by CT, and another patient was CT positive but missed the intraventricular hemorrhage by CT. In the control population (n=18), 61% were male and 39% female, and the average patient age was 34.83±14.30 years. There was no age difference between patients and controls, but a significant gender difference was found between these two groups (p=0.002, Chi-square test).

**Table 1:** Individual demographic and clinical data of the 9 patients enrolled in the study.

**Patient no.**	**Age/ Gender**	**Race**	**GCS**	**LOC**	**Mechanism of Injury**	**SAC**	**CT**	**Structural MRI**
P-001	56/M	Asian	15	1 min	MVA		Negative	Nonspecific WM hyperintensies, Small foci of intraventricular blood on the left. Small blood product in the left lingual gyrus
P-002	36/F	Black	15	10 min	MVA	23	Negative	Negative
P-003	19/M	Black	15	2 min	MVA	25	Negative	Nonspecific FLAIR hyerintensity in posterior cerebral WM
P-004	35/M	Arabic	15	30 min	Assault	26	Positive hemorrhagic contusion on left parieto-temporal lobe	Hemorrhagic contusion on left parieto-temporal lobe, left ventricular hemorrhage
P-005	52/M	Black	15	5 min	Assault	19	Negative	Non-specific, multiple scattered discrete foci in cerebral WM
P-006	53/M	Black	15	5 min	MVA	24	Negative	Non-specific: Two isolated punctate foci of blood in the right periatrial WM
P-007	39/M	Caucasian	15	2 min	Assault	22	Small SAH in right Sylvian Fissure	SAH in right Sylvian Fissure
P-008	58/M	Caucasian	15	2 min	Assault	19	Negative	Non-specific, super sella lesion, congenital cistern lesion in posterial fossa
P-009	23/M	Caucasian	15	30 min	Assault	24	Negative	Negative

### Patients’ Neurocognitive Performance

The mean patients’ SAC score was 22.75 ±SD 2.6. We compared this mean with published normative data of over 568 subjects [54] (mean ± SD, 26.3±2.2). The patients’ mean SAC score was significantly below this published mean score (t(8)=-4.180, p=0.0041). Among each subcategory of SAC test, patients’ delayed recall and immediate memory were both significantly lower than published normalized data (p=0.042 and p=0.021, respectively), and concentration had a trend of reduction towards significance (p=0.056).

### MRI Findings

In structural MRI (T1, T2 FLAIR, and SWI) analysis, there was no group difference in SAC scores between patients with positive anatomical MRI findings and patients with negative anatomical MRI findings. In DTI analysis, both increased and decreased FA beyond the threshold (t>=4) were found in all patients with variable number of clusters in different locations of the WM. By adding these clusters together, the volume of abnormal FA, called “lesion load”, was used to correlate with patients’ neurocognitive and biomarker data. DTI lesion load with pure increased FA was found significantly higher than that with pure decreased FA (student t-test, p=0.034 for TBSS analysis; and p=0.017 for VBA analysis). This suggests the increased FA as the main pathology. 

**Figure 1:**
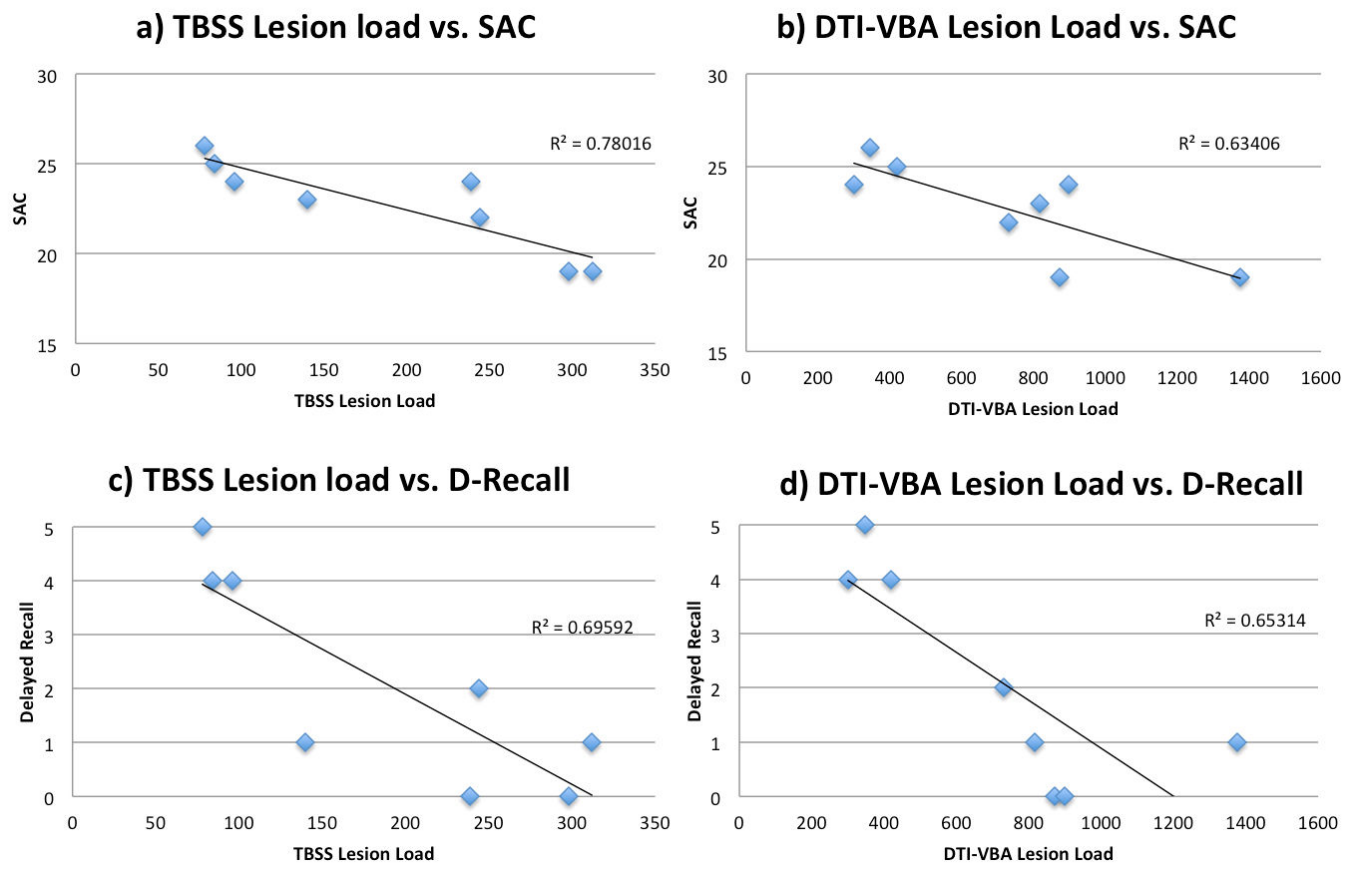
Correlations between DTI lesion load and patients’ neurocognitive data. As demonstrated in the figures, DTI lesion load (both TBSS and VBA data) are significantly correlated with patients’ overall SAC score and delayed recall. R squared values are shown on each figure for linear regression.

Figure 1 presents the relationship between SAC scores and overall DTI lesion load, which contains total number of voxels with abnormal FA (either increased or decreased). Specifically, SAC scores were found to be inversely correlated with DTI-TBSS lesion load (Pearson r=-0.883, p =0.004) and DTI-VBA lesion load (r=-0.796, p=0.018) and had an almost significant correlation with age (r=-0.701, p =0.053). By looking at subcategories of SAC scores, both DTI-TBSS lesion load and DTI-VBA lesion load were correlated with SAC delayed recall (r=-0.834, p=0.010 and r=-0.796, p=0.018, respectively). DTI-TBSS and DTI-VBA lesion loads were strongly correlated (Pearson r=0.881,p=0.002). There was also a partial correlation of SAC scores with DTI-TBSS and DTI-VBA lesion loads after controlling for age (r=-0.893, p=0.0001; r=-0.82, p=0.0016, respectively) and a partial correlation of SAC delayed recall with patients’ DTI-TBSS and VBA lesion loads, controlling for age (r=-0.858, p=0.001; r=-0.811, p=0.002, respectively). Similarly, after controlling for age, DTI-TBSS and DTI-VBA lesion loads remained significantly correlated (Pearson r=0.853 and p=0.001).

By further investigating the FA increase vs. FA decrease in association with patients’ SAC score, the lesion load for pure FA increase was found to be significantly correlated with patients SAC scores (Pearson r=-0.87, p=0.005 for TBSS; and r=-0.86, p=0.005 for VBA) and delayed recall (Pearson r=-0.79, p=0.018 for TBSS and r=-0.770, p=0.025 for VBA). In contrast, the lesion volume for pure FA decrease was neither correlated with SAC nor delayed recall (all p>0.05 for Pearson correlation).

The structural imaging finding, including SWI, was neither correlated with patients’ SAC scores nor PCS scores (all p>0.05).

Given the fact that over half of cases have non-specific findings on structural MRI, particularly WM hyperintensities, student T-tests were further performed to see its effect. No group difference was found between cases with non-specific findings and cases without non-specific findings (all with p>0.3), in terms of their SAC score, DTI lesion loads (both TBSS and VBA data), or biomarker levels (both UCH-L1 and GFAP). 

### Serum Concentrations of UCH-L1 and GFAP

**Figure 2:**
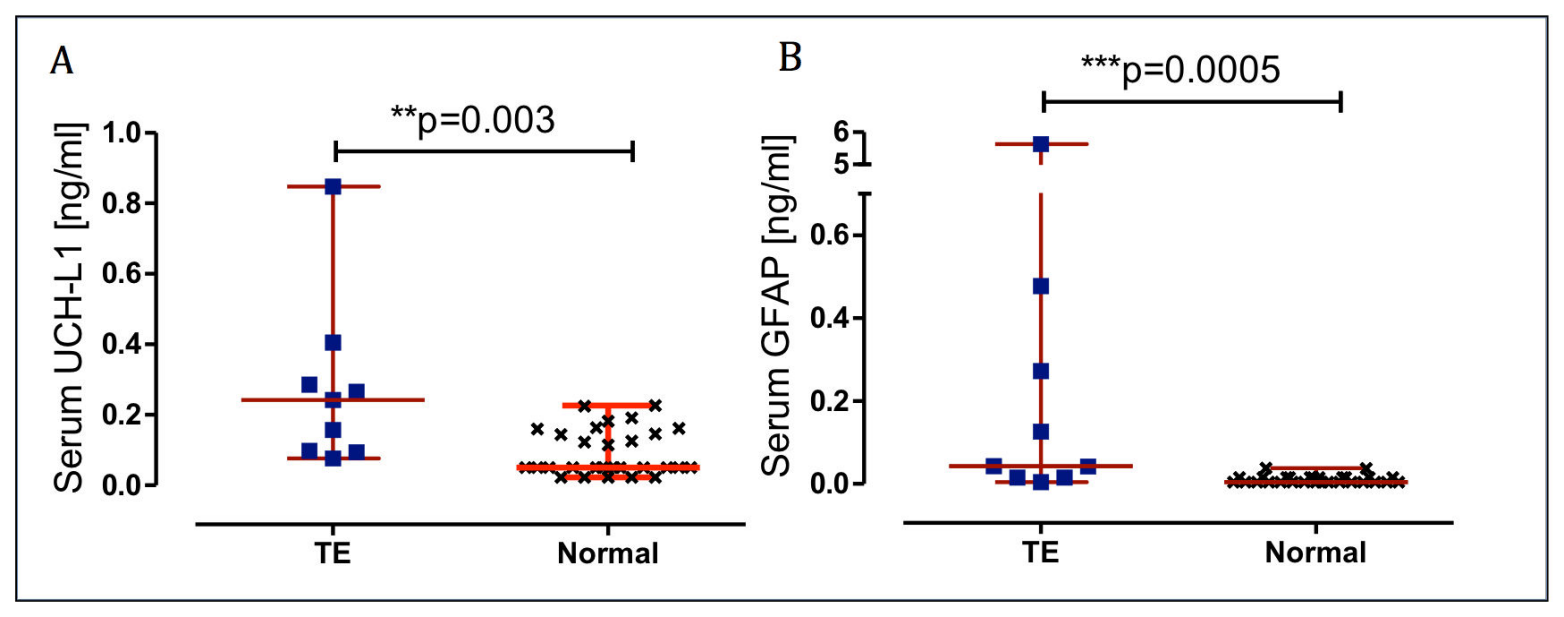
Dot plots demonstrating UCH-L1 and GFAP concentrations. Serum UCH-L1 (A) and GFAP (B) concentrations on admission in TBI patients and in controls. Error bars represent median and range. Significant differences are indicated with ** (P< 0.01) or *** (P <0.001) (Mann–Whitney U-test).

**Figure 3:**
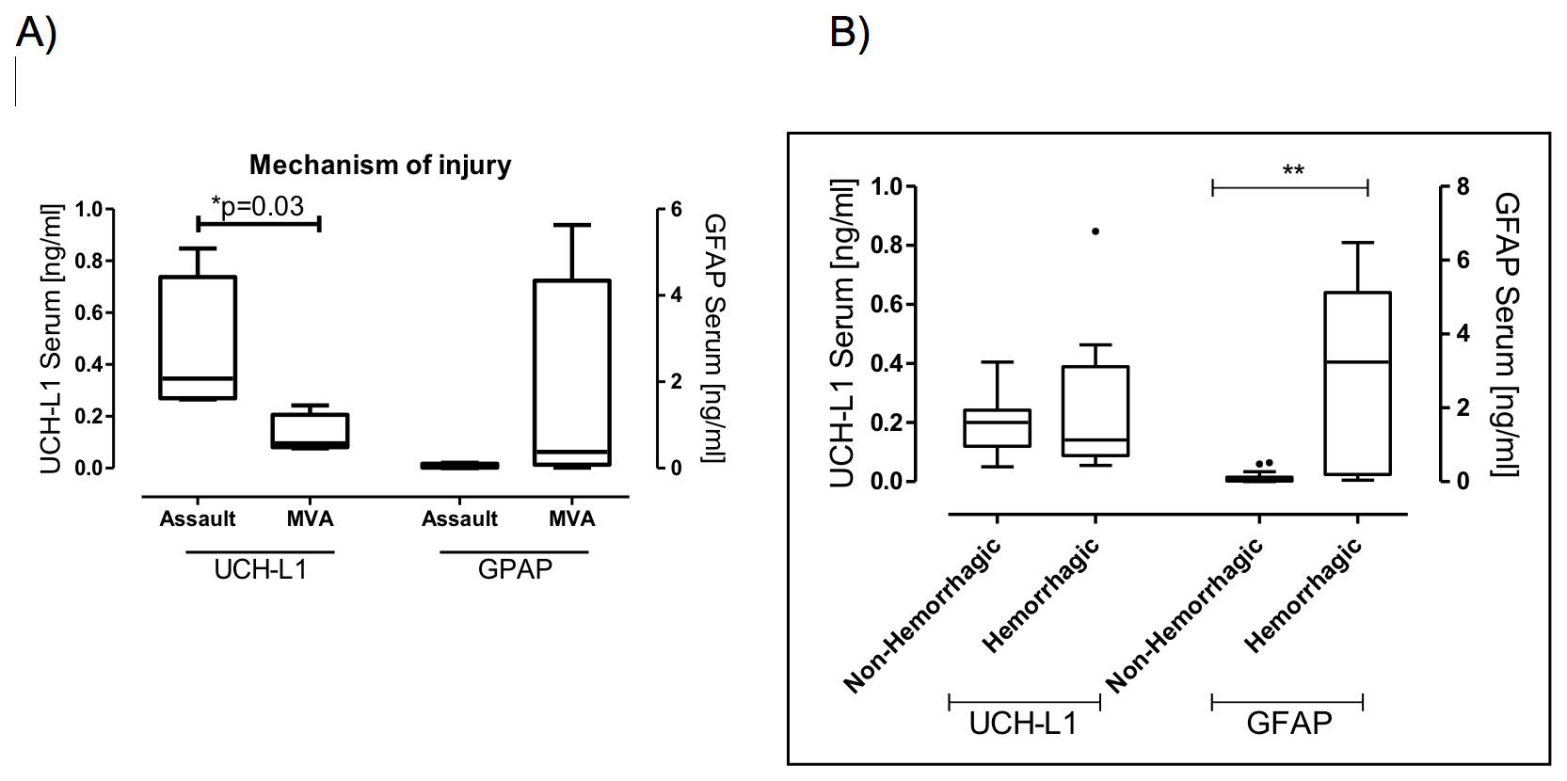
Box-and-whisker plots demonstrating UCH-L1 and GFAP concentrations. (A) Serum UCH-L1 and GFAP concentrations in patients who were victims of assault and in patients injured in a MVA. (B) Serum UCH-L1 and GFAP concentrations in patients with ventricular hemorrhages and hemorrhagic contusions and in patients with non-hemorrhagic lesions. The black horizontal line in each box represents the median, with the boxes representing the interquartile range. Significant differences are indicated * (P< 0.05) or ** (P <0.01) (Mann–Whitney U-test).

Median serum concentrations taken at the time of hospital admission in the patients, within 6 hours after injury, were raised 4.9 folds for UCH-L1, and 10.6 folds for GFAP compared to the laboratory reference values in controls (see Table 2 and Figure 2). Serum UCH-L1 concentrations on admission did not correlate with GFAP (r=-0.24, p=0.52). Serum biomarker concentrations at the time of hospital admission did not correlate with age, time to sample withdrawal, GCS, duration of LOC, SAC or PCS score (all p>0.5). Patients injured in assault had significantly higher UCH-L1 concentrations than patients injured in a MVA (median 0.35 vs 0.10 ng/ml, p=0.03) (Figure 3) while GFAP concentrations were not associated with mechanism of injury. 

**Table 2:** Serum concentration of UCH-L1 and GFAP in patients with mTBI and in controls.

		**Serum UCH-L1 (ng/mL**)	**Serum GFAP (ng/mL**)
	**Admission**	0.242 (0.096-0.336)*	0.043 (0.015-0.375)*
**TBI**	**Hemorrhagic**	0.164(0.098-0.314)*	0.517(0.239-4.610)** †
	**Non-Hemorrhagic**	0.171(0.107-0.248)**	0.015(0.015-0.06)**
**Controls**		0.05 (0.05-0.153)	0.004 (0.004-0.015)

Data are given as median (interquartile range).

* p <.01 and ** p<.001 (p values of the Mann-Whitney test for differences between the groups [TBI versus Controls])

† p<.01 (p values of the Mann-Whitney test for differences between the groups [Hemorrhagic versus Non-Hemorrhagic])

Neither UCH-L1 nor GFAP concentrations were associated with structural MRI grading or DTI lesion load, as assessed by Pearson correlation (p>0.05). However, patients with hemorrhages on structural MRI presented significantly higher levels of GFAP compared with the non- hemorrhagic group (p=0.002) (see Table 2 and Figure 3).

Temporal profile of biomarker levels indicates that UCH-L1 tend to peak at the admission (within 6 hours after injury) and GFAP at 12 hours after injury. See supplement Figure S1 for details.

### Length of Loss of Consciousness

All patients had variable length of loss of consciousness (LOC). While length of LOC has often been considered as an injury severity measure, we did not find any correlation between LOC length and any other variables, including SAC, structural MRI, DTI lesion load, and blood biomarkers (Pearson correlation p>0.05).

### Illustrative Cases

#### Case 1 - Intraventricular Hemorrhage Missed by CT

**Figure 4:**
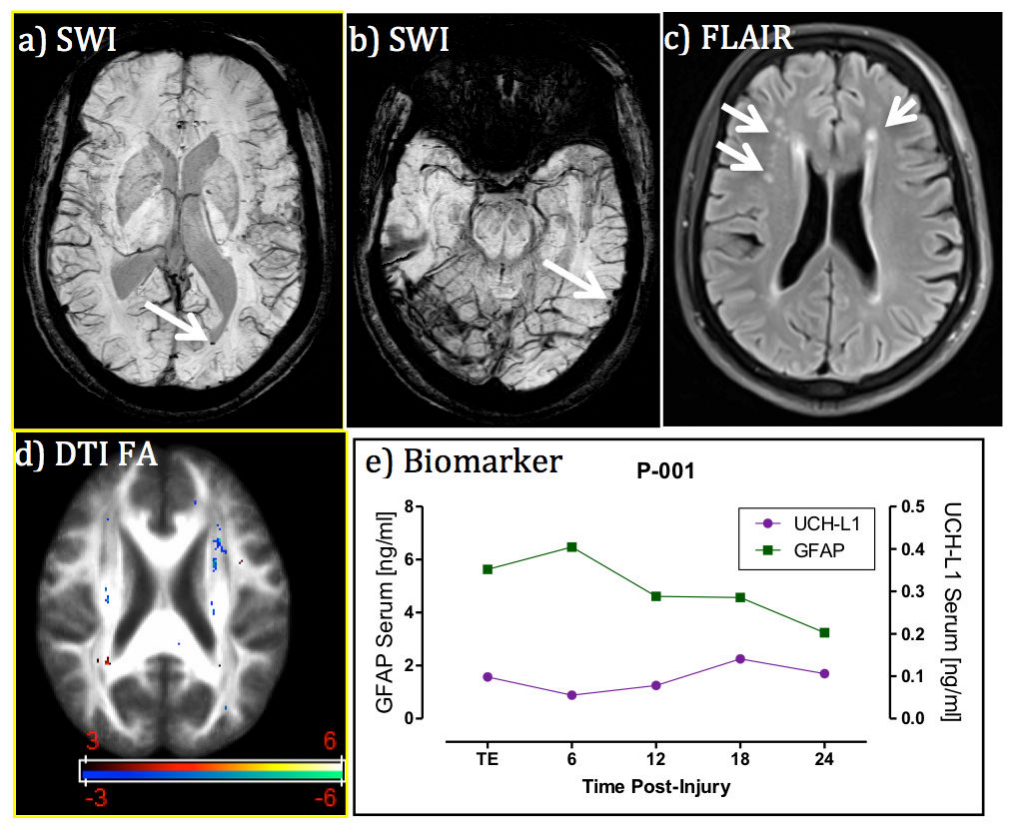
Case 1. MRI and biomarker profile in a patient with intraventricular hemorrhage missed by CT. Panels **a**) and **b**) are SWI images at different locations of the brain showing intra-ventricular blood and left lingual gyrus blood product (see arrows); panel **c**) is FLAIR image showing non-specific white matter hyper- intensities (see arrows); panel **d**) is DTI FA map showing the co-existence of voxels with increased and decreased FA measures (red color means FA decrease and blue color FA increase in comparison with controls, t>3 for t-test); and panel **e**) is blood biomarker temporal profile, which exhibiting extraordinarily high GFAP levels over time in comparison with controls (median 0.004, interquartile range 0.004-0.015). Despite being missed by CT, the injury was still detected by both blood biomarker and MRI.

A 56-year old male driver suffered mental status change after his car was rear-ended by another vehicle. He presented in the ED with a GCS score of 15 with abrasion and a small laceration on his left eyebrow without closure and left clavicle fracture. His major clinical symptoms were left shoulder pain and headache. Non-contrast CT scan showed no intracranial abnormalities. Initial MRI scanning performed at 20 hours after injury revealed small foci of intraventricular blood on the left side, small blood product in the left lingual gyrus seen on SWI images and several nonspecific WM hyperintensies on FLAIR images (Figure 4). Graphs displaying time course of UCH-L1 and GFAP are shown in Figure 4. In the sample obtained on admission, GFAP levels were markedly high. GFAP elevation persisted throughout the monitoring time gradually decreasing at 24 hours post injury (median 4.610, range 3.241–6.475 ng/ml). In contrast, in the same blood samples, UCH-L1 levels were only slightly high compared to controls (median 0.098, range 0.055–0.1410 ng/ml). 

#### Case 2 – Traumatic axonal Injury case with normal structural MRI and high UCH-L1 level

**Figure 5:**
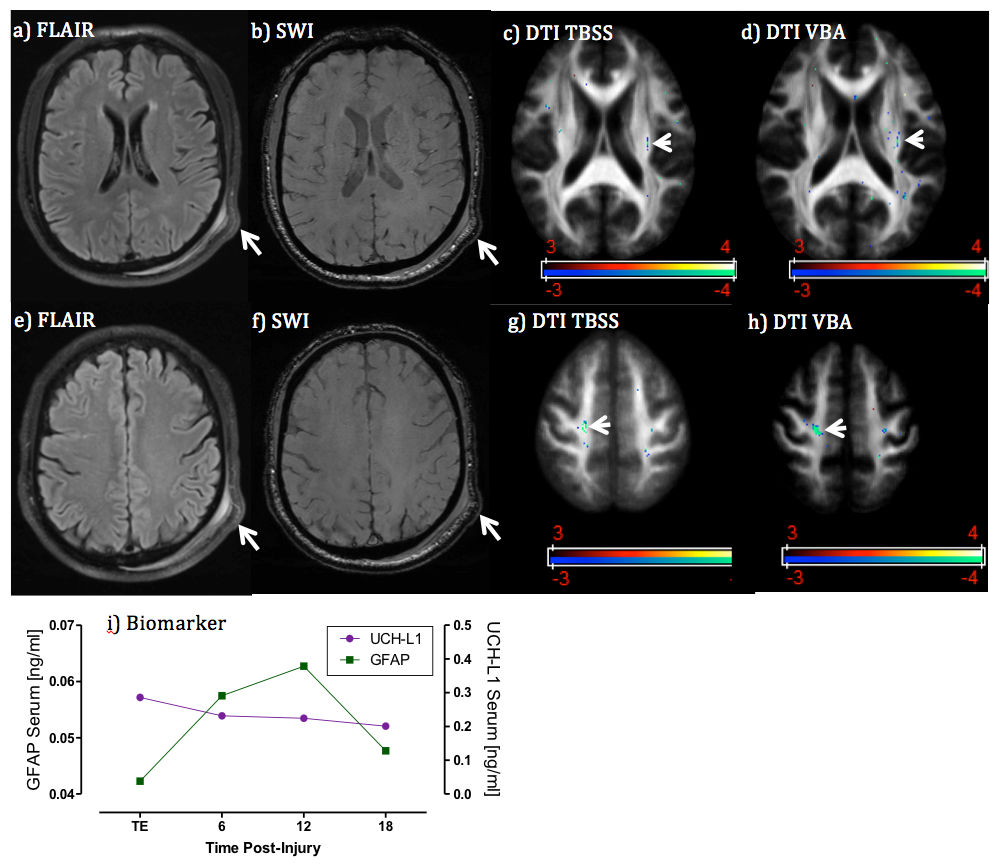
Case 2. MRI and biomarker profile in a patient with traumatic axonal injury but normal-appearing structural MRI. Panels **a-d**) are MRI images at the corpus callosum and fornix level. Panels e-h) are MRI images at the level of superior coronal radiata. Panel **i**) is blood biomarker profile. FLAIR and SWI images both indicate the skalp contusion at the parieto-occipital region (long arrows) but normal-appearing brain structure. However, both DTI TBSS and VBA analyses detected significantly reduced FA values at the ipsilateral side (corticospinal tract) and contralateral side (superior corona radiata) of brain white matter (arrow heads), in suggestion of coup and contra coup injury at the microstructure of white matter. Cold color indicates reduced FA values in comparison with controls (t>3 for t-test). Blood biomarkers indicate slightly increased GFAP levels over time but significantly increased UCH-L1 at the admission.

This 64-year old male patient was a victim of an assault and suffered brief loss of consciousness and femur fracture. He presented in the ED with a GCS score of 15 with symptoms of severe headache, dizziness, not feeling sharp, memory problems, poor concentration, fatigue/sluggish, sadness/depression, and irritability. His SAC score was 19 out of 30, and delayed recall 0 out of 5. Non-contrast CT scan showed no intracranial abnormalities. MRI scan performed at 7 hours after injury demonstrated multiple foci of non-specific WM hyper-intensities on FLAIR, but no intracranial bleeding on SWI. DTI data revealed multi-clusters with significantly decreased FA in the superior corona radiata and corticospinal tract (see Figure 5); these findings were suggestive of traumatic axonal injury at microstructural level. The results of neurochemical of biomarker time course demonstrated UCH-L1 being consistently elevated (>0.2ng/ml) with high initial values that gradually decreased. GFAP was slightly increased with a peak at 12 hours post-injury (peak 0.063 ng/ml). 

## Discussion

To our knowledge, this is the first effort to combine both blood biomarker and advanced MRI to improve the detection and characterization of mild TBI in the acute setting (within 24 hours after injury). We found that a) the biomarker levels were significantly higher in mTBI patients after injury; b) the levels of GFAP were highest in all subjects with intracranial bleeding on SWI, which is new finding in mTBI research; c) the total volume of WM voxels with abnormal DTI FA measures is correlated with patient’s neurocognitive status, including memory; and d) DTI FA values could both increase and decrease in the acute setting, which is also a new finding in mTBI research. In the acute setting, the immediate challenge for emergency physicians is to identify those CT negative but symptomatic patients with intracranial abnormalities that may be predictive of long term neurocognitive sequalae [12]. Given the fact that most mTBI patients stay in the emergency department from only a few hours to 24 hours’ observation, our comprehensive approach at this stage, while the patients are still in the emergency department, is more likely to help emergency physicians make decisions on patient management. 

This study also ideally extends the previous work by our co-authors demonstrating the relationships between different pathways for UCH-L1 and GFAP and different types of brain injury pathophysiology after severe TBI as characterized by CT [55]. In addition to the previous findings, these pilot data suggest that the combined use of biochemical markers and advanced MRI techniques may provide an important tool to evaluate and characterize mTBI patients, which is of importance for the understanding of the different pathophysiological mechanisms following TBI and for the development of effective therapies.

### The heterogeneity of brain injury pathology

It is well recognized that brain injury pathology is heterogeneous and complex [56]. Each technique employed in this study brings unique aspects of brain injury pathology and contributes to the whole picture: intracranial bleeding, detected by SWI, manifests blood vessel damage [25]; DTI finding signifies the damage of WM integrity [13,14]; UCH-L1 for neuronal injury [44,57]; and GFAP for glial damage [55,58]. These different pathologies may be correlated with each other and, together, they can cover the spectrum of brain injury that contributes to impaired brain function. Our data demonstrated that intracranial bleeding was associated with elevated GFAP levels, which validated our first hypothesis regarding the association between GFAP and structural MRI. It also suggests an association between glial injury and vascular damage in mild TBI. Meanwhile, the non-association between UCH-L1 and MRI data goes against our second hypothesis on the relationship between UCH-L1 and DTI findings. As a matter of fact, both imaging and biochemical markers demonstrated abnormalities of mTBI in different aspects, suggesting that they are also complementary to each other for brain injury detection. This further confirms the heterogeneity of brain injury pathology.

### Intracranial bleeding and elevated GFAP levels

Searching for intracranial bleeding is critically important in diagnostic radiology. Despite the fact that most mild TBI patients have negative CT findings, those mTBI patients (GCS 13-15) with positive CT findings are classified as “complicated mild TBI” [59]. As a subcategory of mTBI, their outcome tend to be worse than other mTBI patients with negative CT findings and even close to moderate level of TBI [59]. Regarding the role of structural MRI findings on mTBI classification, a recent study of 135 mTBI patients, scanned at 12 days after injury, demonstrated that one or more brain contusions on structural MRI, and ≥4 foci of hemorrhagic axonal injury on MRI, were each independently associated with poorer 3-month outcome [60]. 

From pathophysiological perspective, GFAP is a structural protein of astroglial cells that are located in the intracellular space of astrocytes. The damage to astrocytes will cause the release of GFAP into extra-cellular space and that might leak into the blood stream through a compromised blood-brain barrier (BBB) [61]. Furthermore, the end processes of astrocytes surround the endothelial cells of vasculature system and make astrocytes an integral part of neural vascular unit [62]. The damage or temporal opening of the BBB will also likely further damage to the surrounding astrocytes as well. Supporting this, in stroke studies, considerable amount of data demonstrated significantly increases in GFAP in expanding intra-cerebral hemorrhage (ICH) than that in ischemic stroke [63,64]. Other studies reported a close correlation between GFAP serum concentration and ICH volume [65,66]. Even a multi-center clinical trial was conducted to use GFAP to differential ICH from ischemic stroke [65]. In addition to ICH, our data demonstrated that all intracranial hemorrhage cases, including both extra-axial and parenchymal hemorrhage, have significantly elevated GFAP levels. This implies that GFAP levels in blood serum have the potential to serve as a quick screening biomarker to triage mTBI patients for MRI confirmation of intracranial bleeding for prediction of an unfavorable outcome.

### The role of BBB

The elevated biomarker levels measured in our patients support the idea of a BBB breakdown that has often been documented in patients with TBI even after mild injuries [67]. Indeed, both UCH-L1 and GFAP are CNS-specific proteins with very low concentrations in blood in healthy people, almost below the threshold of detection by using current biomarker technology [40,46]. The elevated level of either one requires the same pathway to leak into the blood pool: the compromise or breakdown of the BBB. Given the much smaller size of UHCL-1 and GFAP than red blood cells, these proteins could more easily get into the blood stream through BBB temporal opening. Consequently, the elevated biomarker levels seem to detect a BBB compromise more relevant than the MRI-detectable bleeding. At a microcopic level, the BBB damage may not be severe enough or the temporal opening of BBB may not be long enough to allow enough red blood cells to pass through or cause sufficient amount of leakage that makes it visible as bleeding on neuroimaging at the macro-level [40]. However, this BBB compromise may be already big enough for sufficient amount of small protein biomarkers to leak into the blood stream and become detectable with modern biomarker detection techniques. Compared with detectable bleeding, which consists of only a small fraction of mTBI patients, the elevation of CNS-specific proteins in the blood pool might be able to serve as a more sensitive biomarker for the compromise of BBB in mTBI at the acute stage.

### Correlation with patients’ neurocognitive performance

Our data showed that DTI lesion loads, measured as both TBSS FA and VBA FA lesion loads, are correlated with their SAC score and delayed recall. More evidence reported that DTI FA values are correlated with mTBI patients’ neurocognitive outcome [14,68]. Particularly, certain regions of DTI WM tract are correlated with patients’ specific neurocognitive outcome [14,68]. As an example, Muhkerjee et al reported that DTI findings are correlated with patients’ neurocognitive performance, but not hemorrhage [69,70]. Our DTI finding at the acute stage is in the same line as the published result at the sub-acute or chronic stage. This further confirms the hypothesis that there might be microstructural damages or changes in WM tracts that account for patients’ neurocognitive deficits. However, this small-scale damage may not reach to the degree of vessel rupture that causes bleeding or hemorrhage. 

### DTI FA increase or decrease

In this study, we also noticed the co-existence of both increased and decreased FA values in mTBI patients within 24 hours after injury and the dominance of increased FA lesions. All DTI studies of moderate to severe TBI patients [23,71-73] and subacute/chronic mTBI patients [22,69,74-76] report FA *decreases* which are correlated with clinical or neuropsychological measures. However, there are seemingly contradictory findings in mild TBI in the acute stage (within one week after injury) in the literature: Inglese [22] and Arfanakis[21] both reported FA *decreases*, while Wilde [77], Bazarian [78], and Mayer [79] reported FA *increases* and decreased radial diffusivity. Furthermore, Michael Lipton et al [80] reported bi-directional changes (both increase and decrease) of FA in chronic mTBI patients. Most recently, Bazarian et al [81] studied 9 high school athletes with diagnosed concussion or multiple sub-concussive blows and also reported bi-directional changes of FA at chronic stages. Of particular note, the terminology “acute stage” could mean quite different timing frames across the studies: some defined it as within 24 hours after injury and some even as within 7 days after injury. To date, only two studies reported MRI scan of mTBI patients within 24 hours after injury and they both have only a handful of patients [21,77]. The co-existence of both FA decrease and increase within 24 hours after injury is a relatively new in the field and needs further investigation in a relative large number of patients. It further demonstrates the heterogeneity of mTBI pathology at this stage. Meanwhile, lesion load with increased FA is significantly higher than the lesion load with decreased FA in our study. Increased FA lesion load, but not decreased FA lesion load, is correlated with patients’ SAC and delayed recall scores in our data. It has been suggested that increased FA *acutely* may reflect cytotoxic edema [77], which would shunt extracellular fluid into swollen cells. This could have the effect of reducing inter-axonal free water and therefore reducing radial diffusivity and increasing overall fractional anisotropy. In contrast, a decreased FA could be due to several possible mechanisms: a) an impaired axonal transportation or axonal swelling, both of which cause decrease of longitudinal diffusivity along white matter tract; and b) vasogenic edema, which causes an enlarged extra-cellular space and an increased radial diffusivity. Our data of bi-directional changes of FA and the dominance of FA increase demonstrates the heterogeneous pathology while cytotoxic edema might be a leading cause for patients’ neurocognitive symptoms at hyper-acute stage (within 24 hours after injury).

### The need for an axonal injury biomarker

Our findings confirm that a panel of biomarkers rather than a single analyte seem to have the most utility for the diagnosis of mTBI patients, and improved characterization of the injury. Importantly, in the current study neither UCH-L1 nor GFAP was associated with WM injury identified by DTI. Because traumatic axonal injury is believed to be a major determinant of functional and neurocognitive symptoms following TBI as demonstrated by the correlation between DTI and patients’ neurocognitive deficits, there might be a need for specific axonal injury biomarkers. Further work is needed to develop additional biomarker platforms, including axonal injury markers, in addition to the neuronal and glial damage proteins examined here, and to identify the relationships with advanced MRI techniques and patient outcomes that will help validate and confirm their clinical utilities in the acute setting [12]. 

### Limitations and future work

Despite its encouraging finding, this preliminary work has limitations, including a small sample size and the lack of long-term outcome data. It has been reported that DTI FA abnormality in certain WM tracts is correlated with patients’ specific neurocognitive deficits in TBI [14,68]. This correlation between DTI lesion load and SAC score is consistent with the literature. However, the findings in our study with relative small sample size need further validation in a large number of patients. Further, it has been reported that orthopedic injury cases could also have slightly increased GFAP levels [47]. Though not significantly higher than controls, this could be another confounding factor which makes blood biomarker non-specific to brain injury. Therefore, a cohort of orthopedic injury controls is also needed for this study validation. Another confounding factor is that the mTBI patients and healthy controls are not age-matched. As demonstrated in our data, age is also correlated with patients’ neurocognitive score. A demographically (included age, gender and education) matched study in future will be able to eliminate this confounding factor. In short, additional research will be required to validate our current findings in a large cohort of patients and demographically matched controls with longitudinal follow up and to further determine the relationships between neuroimaging and biomarker findings in the prediction of mTBI outcome.

## Conclusions

To summarize, this work represents the first effort of combining both blood protein biomarkers and advanced MRI to improve the detection and characterization of brain injuries after mild TBI in the acute stage (within 24 hours after injury). Our data demonstrate elevated GFAP and UCH-L1 levels in mTBI patients at the acute stage in comparison with controls. Particularly, all cases with intra-cranial hemorrhage had significantly higher GFAP levels than those without hemorrhage. Patients’ DTI measures were correlated with their neurocognitive status at this stage. This overlapping and complementary role of blood biomarkers and imaging in brain injury detection offers the promise that they might be used in conjunction in the management of patients with mTBI. Further studies with larger numbers of patients will be required to assess the reproducibility of these findings and to confirm the potential clinical utilities as diagnostic adjuncts in the acute setting.

## Supporting Information

Figure S1 **Serum biomarker levels over the first 24 hours after mTBI compared with controls.** Serum UCH-L1 (A) levels are maximal early after injury (on admission) (TE=0.24 [0.096-0.346]), while GFAP (B) concentrations peaked 12 hours after injury (0.35 [0.036-2.56]). Error bars represent median and IQR.
(TIF)
