# Supplementary figures and images for: Combining Biochemical and Imaging Markers to Improve Diagnosis and Characterization of Mild Traumatic Brain Injury in the Acute Setting: Results from a Pilot Study
Source: PLoS One. 2013 Nov 19;8(11):e80296. doi: 10.1371/journal.pone.0080296 (PMC3833898; doi:10.1371/journal.pone.0080296)

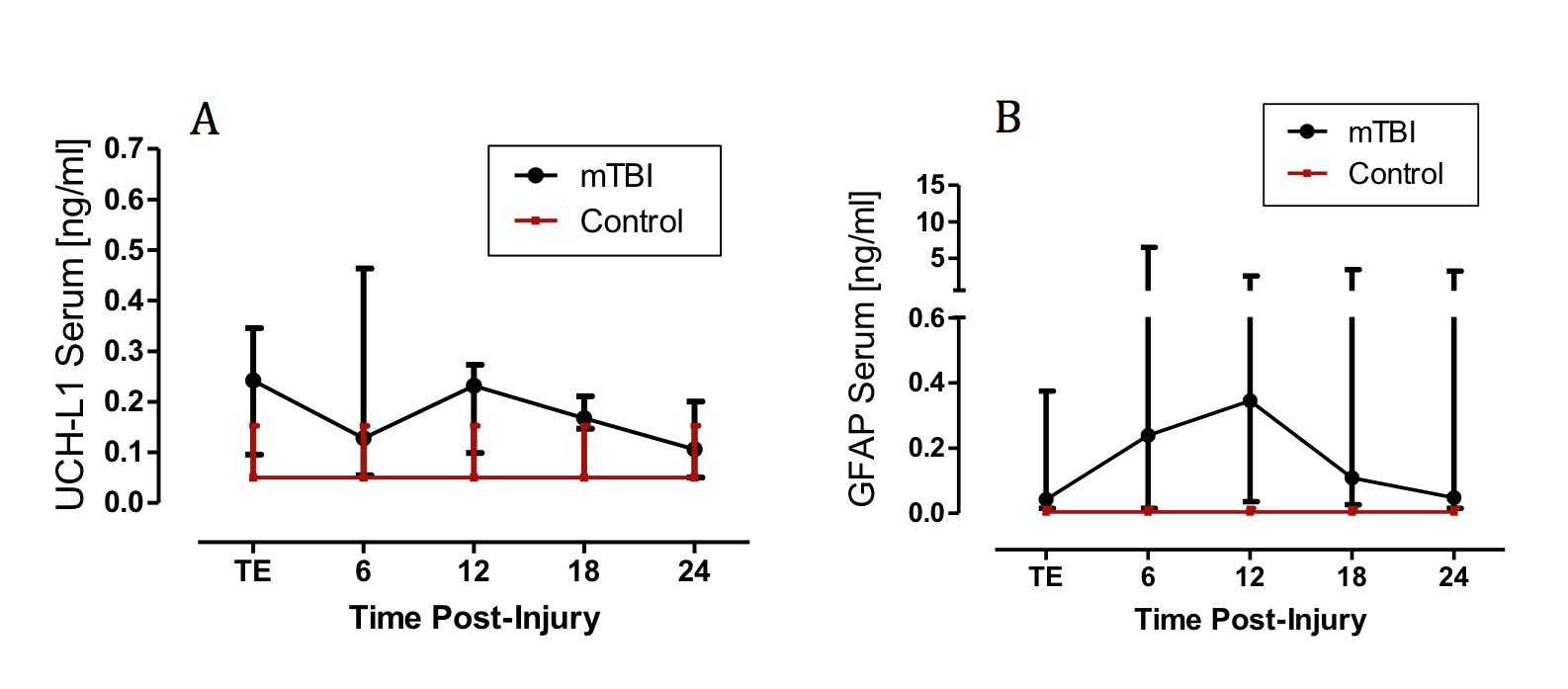

Supplement: Figure S1 — Serum biomarker levels over the first 24 hours after mTBI compared with controls. Serum UCH-L1 (A) levels are maximal early after injury (on admission) (TE=0.24 [0.096-0.346]), while GFAP (B) concentrations peaked 12 hours after injury (0.35 [0.036-2.56]). Error bars represent median and IQR. (TIF) [file pone.0080296.s001.tif]
